# Improved outcomes of cardiac resynchronization therapy with a defibrillator in systolic heart failure: Analysis of the Japan cardiac device treatment registry database

**DOI:** 10.1002/joa3.12952

**Published:** 2023-11-14

**Authors:** Hisashi Yokoshiki, Akihiko Shimizu, Takeshi Mitsuhashi, Kohei Ishibashi, Tomoyuki Kabutoya, Yasuhiro Yoshiga, Yusuke Kondo, Haruhiko Abe, Wataru Shimizu, Members of the Implantable Cardioverter‐Defibrillator (ICD) Committee of the Japanese Heart Rhythm Society

**Affiliations:** ^1^ Department of Cardiovascular Medicine Sapporo City General Hospital Sapporo Japan; ^2^ UBE Kohsan Central Hospital Ube Japan; ^3^ Department of Cardiovascular Medicine Hoshi General Hospital Koriyama Japan; ^4^ Department of Cardiovascular Medicine National Cerebral and Cardiovascular Center Suita Japan; ^5^ Division of Cardiovascular Medicine, Department of Medicine Jichi Medical University School of Medicine Shimotsuke Japan; ^6^ Division of Cardiology, Department of Medicine and Clinical Science Yamaguchi University Graduate School of Medicine Ube Japan; ^7^ Department of Cardiovascular Medicine Chiba University Graduate School of Medicine Chiba Japan; ^8^ Department of Heart Rhythm Management University of Occupational and Environmental Health Kitakyushu Japan; ^9^ Department of Cardiovascular Medicine Nippon Medical School Bunkyo‐ku Japan

**Keywords:** cardiac resynchronization therapy with a defibrillator (CRT‐D), ICD therapy, implantable cardioverter–defibrillator (ICD), systolic heart failure, the Japan cardiac device treatment registry (JCDTR) database

## Abstract

**Background:**

Temporal change in outcomes of heart failure patients receiving cardiac resynchronization therapy with a defibrillator (CRT‐D) is unknown.

**Methods:**

We assess outcomes and underlying heart diseases of patients receiving CRT‐D with analyzing database of the Japan cardiac device treatment registry (JCDTR) at the implantation year 2011–2015 and New JCDTR at the implantation year 2018–2021.

**Results:**

Proportion of nonischemic heart diseases was about 70% in both the groups (JCDTR: 69%; New JCDTR: 72%). Cardiac sarcoidosis increased with the rate of 5% in the JCDTR to 9% in the New JCDTR group. During an average follow‐up of 21 months, death from any cause occurred in 167 of 906 patients in the JCDTR group (18%) and 79 of 611 patients in the New JCDTR group (13%) (adjusted hazard ratio [aHR] in the New JCDTR group, 0.72; 95% confidence interval [CI]: 0.55–0.94; *p* = .017). The superiority was mainly driven by reduction in the risk of noncardiac death. With regard to appropriate and inappropriate implantable cardioverter–defibrillator (ICD) therapy, there was a significant reduction in the New JCDTR group versus the JCDTR group (aHR in the New JCDTR group, 0.76; 95% CI: 0.59–0.98; *p* = .032 for appropriate ICD therapy; aHR in the New JCDTR group, 0.24; 95% CI: 0.12–0.50; *p* < .0001 for inappropriate ICD therapy).

**Conclusions:**

All‐cause mortality was reduced in CRT‐D patients implanted during 2018–2021 compared to those during 2011–2015, with a significant reduction in noncardiac death.

## Graphical abstract

Death from any cause was reduced in CRT‐D patients implanted during 2018–2021 (New JCDTR) compared to those during 2011–2015 (JCDTR) (adjusted hazard ratio [aHR] in the New JCDTR group, 0.72; 95% confidence interval [CI], 0.55–0.94; *p* = .017), with a significant reduction in noncardiac death. There was a significant reduction of appropriate and inappropriate ICD therapy in the New JCDTR group versus the JCDTR group.

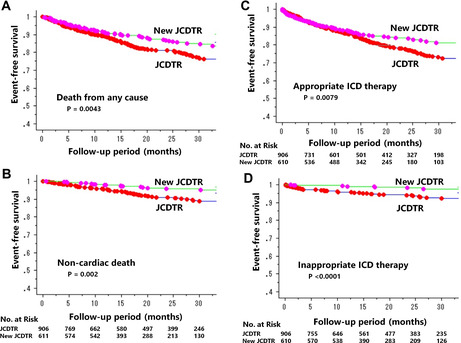

## 1 INTRODUCTION

Cardiac resynchronization therapy (CRT) resulted in clinical improvement of symptomatic heart failure patients associated with a left ventricular ejection fraction (LVEF) of 35% or less and QRS width of 130 ms or more in an initial study in 2002.^1^ With subsequent landmark trials,^2^, ^3^, ^4^, ^5^ the benefit is the greatest in patients with left bundle branch block (LBBB)‐type morphology and QRS width ≥150 ms.^6^, ^7^, ^8^, ^9^, ^10^ In this regard, American and European recommendations for CRT implants were updated with much emphasis on the morphology and interval of QRS in 2013,^11^, ^12^ and a similar revision in Japan was implemented in 2019,^13^ which is likely to affect the characteristics and outcomes of patients receiving CRT devices. The present study aimed to evaluate the latest clinical practice in patients receiving CRT with a defibrillator (CRT‐D) as compared with the earlier cohort with an analysis of the Japan cardiac device treatment registry (JCDTR) versus New JCDTR database.

## 2 METHODS

### 2.1 Study population

The JCDTR and New JCDTR was established in 2006 and 2018, respectively, by the Japanese Heart Rhythm Society (JHRS) for a survey of actual conditions in patients undergoing de novo implantation of cardiac implantable electronic devices (CIEDs) including implantable cardioverter–defibrillator (ICD)/CRT‐D/CRT with a pacemaker (CRT‐P).^14^, ^15^, ^16^, ^17^ A new system, called New JCDTR 2023, started on April 2023, in which data of patients at the implantation date after April 2023 are encouraged to register (https://new.jhrs.or.jp/contents_web/new_jcdtr/ accessed on August 27, 2023). The protocol for the research project has been approved by a suitably constituted Ethics Committee at each institution and it conforms to the provisions of the Declaration of Helsinki.

### 2.2 Device programming

In general, device programming was as follows. VF zone detected ventricular events faster than 185–200 beats/min with at least one train of antitachycardia pacing (ATP) before shock, and the VT zone detected ventricular events faster than 150–170 beats/min with at least three trains of ATP before shock. After the multicenter automatic defibrillator implantation trial‐reduce inappropriate therapy (MADIT‐RIT) trial was published in 2012, the VF zone ≧200–250 beats/min with ATP plus shock and VT zone ≧170 beats/min with delayed therapy (a 60‐s delay), or only monitoring were recommended for primary prevention of sudden cardiac death.^18^ The discrimination algorithms were used at the physician's discretion.

### 2.3 Outcome

Analyzed events were (a) death from any cause; (b) death by heart failure; (c) sudden cardiac death; (d) noncardiac death; and (e) appropriate and (f) inappropriate ICD therapy. Appropriate ICD therapy was defined as an ATP or shock for tachyarrhythmia determined to be either ventricular tachycardia (VT) or ventricular fibrillation (VF). The diagnosis was made by attending physicians.

### 2.4 Statistical analysis

All data are expressed as mean ± SD. Simple between‐group analysis was conducted using Student's *t*‐test. Categorical variables were compared using the chi‐squared test or Fisher's exact test. The Kaplan–Meier curves were constructed to estimate event‐free outcomes in the study groups with comparison using the log‐rank test. Hazard ratios for events in CRT‐D patients of the New JCDTR group versus the JCDTR group were computed with a multivariate Cox proportional‐hazards regression model after adjusting for confounding factors including age, gender, indication, etiology, LVEF, NYHA class, QRS width, and hemoglobin and creatinine levels. Differences with *p* < .05 were considered significant. StatView version 5.0 for Windows (SAS Institute Inc., Cary, NC, USA) or R software ver.3.6.3 (https://www.r‐project.org/) was used for all statistical analyses.

## 3 RESULTS

### 3.1 CRT‐D patients in the JCDTR and new JCDTR

There were 3889 consecutive CRT‐D patients registered in the JCDTR with the implant date from January 2011 to August 2015. The follow‐up data were available in 906 patients as of September 16, 2015 (the follow‐up rate: 23%; the JCDTR group). After 2018, there were 2437 consecutive CRT‐D patients registered in the New JCDTR with the implant date from January 2018 to October 2021. The follow‐up data were available in 611 patients as of April 30, 2022 (the follow‐up rate: 25%; the New JCDTR group).

As compared with the JCDTR group, CRT‐D patients in the New JCDTR group were older with more prolonged QRS width and a tendency of less severe heart failure in terms of NYHA class. Those in the New JCDTR group had more comorbidities such as hypertension, dyslipidemia, and hyperuricemia. Hemoglobin and creatinine level was higher in the New JCDTR than in the JCDTR group. With regard to gender, indication (i.e., primary vs. secondary prevention of sudden cardiac death), etiology (i.e., ischemic vs. nonischemic), LVEF, and BNP level, there was no significant difference between the two groups (Table 1).

**TABLE 1:** Characteristics of the patients.

	JCDTR (*n* = 906)	New JCDTR (*n* = 611)	*p* value
Age (years)	66.5 ± 11.2	68.4 ± 11.0	.0011
Male	689 (76.0)	469 (76.7)	.74
Primary prevention	620 (68.4)	443 (72.5)	.089
Underlying heart disease			.22
Ischemic	277 (30.6)	169 (27.7)	
Nonischemic	629 (69.4)	442 (72.3)	
LVEF (%)	27.3 ± 9.6	27.6 ± 8.6	.47
NYHA class			.054
I	37 (4.1)	22 (3.6)	
II	267 (29.5)	218 (35.7)	
III	509 (56.2)	323 (52.9)	
IV	93 (10.3)	48 (7.9)	
Heart rate (/min)	70.3 ± 16.9	69.3 ± 15.9	.23
QRS duration (ms)	152.4 ± 31.7	157.7 ± 28.9	.001
QT interval (ms)	456.6 ± 55.4	471.4 ± 52.2	<.0001
Cardio‐thoracic ratio (%)	58.9 ± 6.6	57.0 ± 6.3	<.0001
Atrial lead			.0086
Absent	118 (13.0)	53 (8.7)	
Present	788 (87.0)	558 (91.3)	
NSVT^a^	239 (65.8)	368 (60.2)	.08
AF	110 (12.1)	219 (35.8)	<.0001
Diabetes mellitus	281 (31.0)	217 (35.5)	.067
Hypertension	365 (40.3)	291 (47.6)	.0047
Dyslipidemia	271 (29.9)	273 (44.7)	<.0001
Hyperuricemia	172 (19.0)	163 (26.7)	.0004
Cerebral infarction	61 (6.7)	55 (9.0)	.102
Peripheral artery disease	27 (3.0)	32 (5.2)	.025
BNP (pg/mL)^b^	776 ± 1805	736 ± 1135	.66
Hemoglobin (g/dL)	12.7 ± 2.1	13.0 ± 2.1	.0064
Creatinine (mg/dL)	1.46 ± 1.52	1.64 ± 1.67	.035

*Note*: Values are mean ± SD, or number (%).

Abbreviations: AF, atrial fibrillation; NSVT, nonsustained ventricular tachycardia.

^a^ Information about the presence or absence of NSVT was available for 363 patients registered in the JCDTR and all patients (*n* = 611) in the New JCDTR.

^b^ The value of BNP was unavailable for 216 patients registered in the JCDTR and 124 patients in the New JCDTR.

**FIGURE 1:**
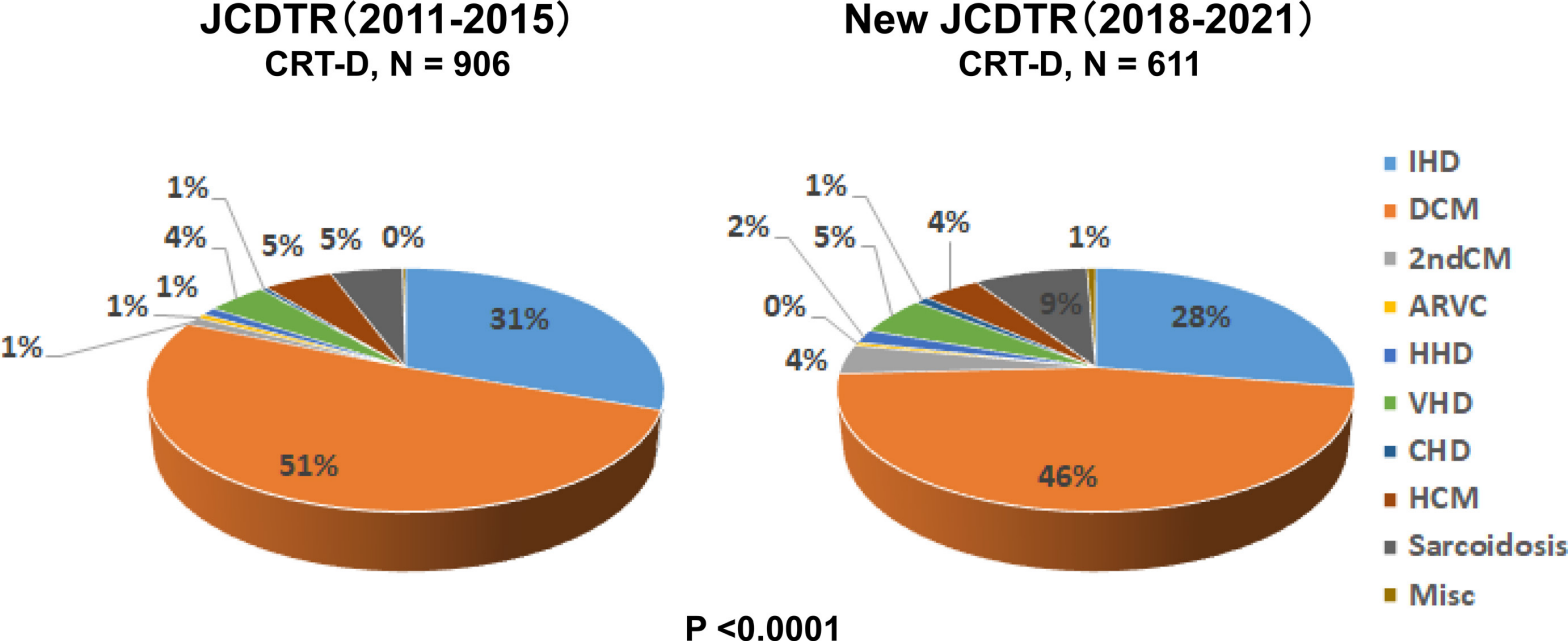
Underlying heart diseases of CRT‐D patients. The percentage is given in a pie chart for CRT‐D patients (*N* = 906) in the JCDTR group from January 2011 through August 2015 and those (*N* = 611) in the New JCDTR group from January 2018 through October 2021. The percentage of underlying heart diseases between two groups was significantly different (*p* < .0001 using the chi‐squared test). ARVC, arrhythmogenic right ventricular cardiomyopathy; CHD, congenital heart disease; DCM, dilated cardiomyopathy; 2nd CM, secondary cardiomyopathy such as amyloidosis, Fabry disease, muscular dystrophy, and post myocarditis; HCM, hypertrophic cardiomyopathy; HHD, hypertensive heart disease; IHD, ischemic heart disease; Misc, miscellaneous; VHD, valvular heart disease.

Proportion of ischemic heart disease was 31% in the JCDTR group and 28% in the New JCDTR, which indicates the predominant etiology of underlying heart diseases was nonischemic (Table 1; Figure 1). Cardiac sarcoidosis increased in the New JCDTR group with the rate of 9% versus 5% in the JCDTR group (*p* < .0001) (Figure 1).

Regarding pharmacological therapy, the use of beta blocker and statin increased, and that of digitalis and oral anticoagulant decreased in the New JCDTR group versus the JCDTR group. There was no difference in the use of angiotensin‐converting enzyme inhibitor (ACEI) or angiotensin II receptor blocker (ARB), mineral corticoid receptor antagonist, and class III anti‐arrhythmic drug between the two groups. Of note, the prescription rate of ACEI/ARB was 66% in the JCDTR group and 63% in the New JCDTR group, which appeared to be low (Table 2).

**TABLE 2:** Pharmacological therapy.

	JCDTR (*n* = 906)	New JCDTR (*n* = 611)	*p* value
Ia	11 (1.2)	7 (1.1)	.90
Ib	26 (2.9)	22 (3.6)	.42
Ic	6 (0.7)	1 (0.2)	.15
Beta blockers	691 (76.3)	508 (83.1)	.0013
III	388 (42.8)	271 (44.4)	.55
Ca^2+^ antagonists	81 (8.9)	46 (7.5)	.33
Digitalis	111 (12.3)	22 (3.6)	<.0001
Diuretics	703 (77.6)	477 (78.1)	.82
ACEI/ARB	601 (66.3)	386 (63.2)	.20
MRA	376 (41.5)	278 (45.5)	.12
Nitrates	96 (10.6)	37 (6.1)	.0022
Statins	295 (32.6)	262 (42.9)	<.0001
Oral anticoagulants	474 (52.3)	264 (43.2)	.0005
Antiplatelet drugs	362 (40.0)	232 (38.0)	.43

*Note*: Data are given as number (%). Ia, Ib, Ic, and III indicates the class Ia, Ib, Ic, and III antiarrhythmic drug, respectively.

Abbreviations: ACEI, angiotensin‐converting enzyme inhibitor; ARB, angiotensin II receptor blocker; MRA, mineralocorticoid receptor antagonist.

### 3.2 Outcomes

**FIGURE 2:**
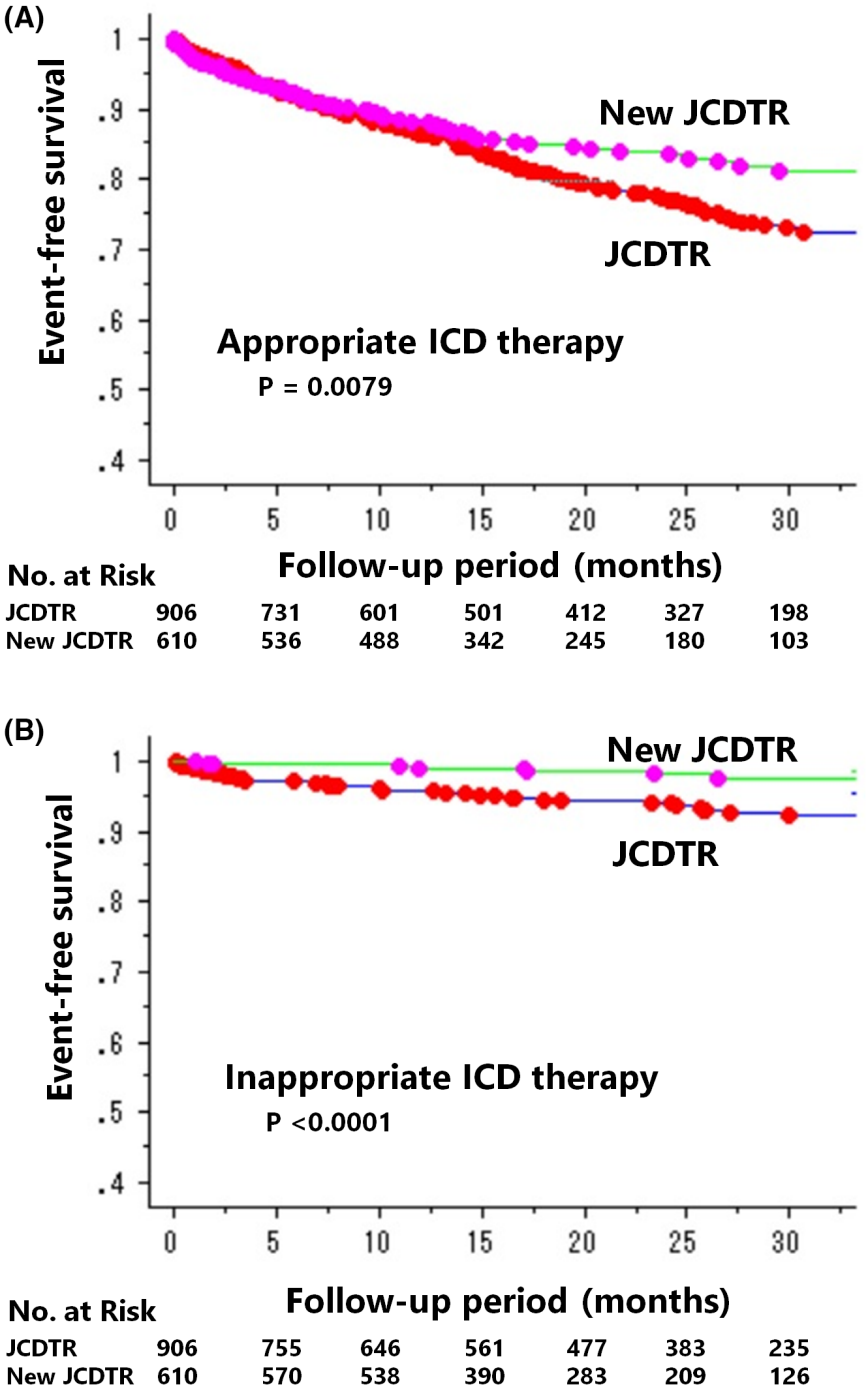
Kaplan–Meier estimates of event‐free survival for appropriate (A) and inappropriate (B) ICD therapy in CRT‐D patients stratified by the New JCDTR group versus the JCDTR group.

Appropriate ICD therapy occurred less frequently in 90 of 610 CRT‐D patients (15%) of the New JCDTR than in 182 of 906 CRT‐D patients (20%) of the JCDTR group during an average follow‐up of 21 ± 10 months and 21 ± 12 months, respectively. The rate was 12% at 1 year and 16% at 2 years in the New JCDTR group, and 13% at 1 year and 23% at 2 years in the JCDTR group (*p* = .0079) (Figure 2A). Similarly, inappropriate ICD therapy occurred less frequently in the New JCDTR (9 of 610: 1.5%) than in the JCDTR group (52 of 906; 5.7%). The rate was 0.9% at 1 year and 1.9% at 2 years in the New JCDTR group, and 4.1% at 1 year and 5.9% at 2 years in the JCDTR group (*p* < .0001) (Figure 2B).

**FIGURE 3:**
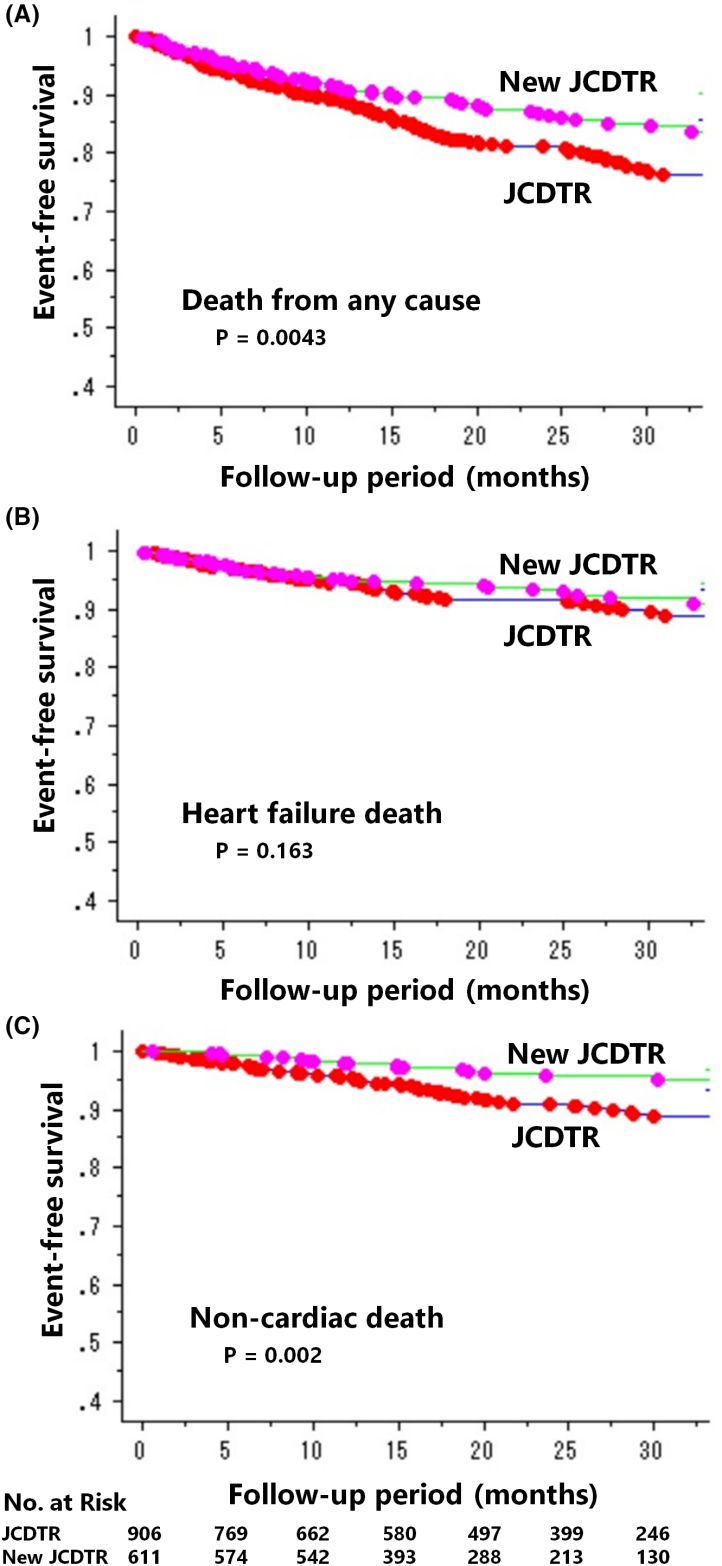
Kaplan–Meier estimates of event‐free survival for death from any cause (A), heart failure death (B), and noncardiac death (C) in CRT‐D patients stratified by the New JCDTR group versus the JCDTR group.

Death from any cause occurred less frequently in 79 of 611 CRT‐D patients (13%) of New JCDTR than in 167 of 906 CRT‐D patients (18%) of the JCDTR group. The rate was 8.9% at 1 year and 13% at 2 years in the New JCDTR group, and 11% at 1 year and 19% at 2 years in the JCDTR group (*p* = .0043) (Figure 3A). With regard to cause of death, there was no significant difference in heart failure death (6.5%: 40 of 611 vs. 8.2%: 75 of 906; *p* = .163) (Figure 3B) and sudden cardiac death (2.3%: 14 of 611 vs. 2.3%: 21 of 906; *p* = .885) between the New JCDTR and JCDTR groups. In contrast, noncardiac death occurred less frequently in 20 of 611 CRT‐D patients (3.3%) of the New JCDTR than in 71 of 906 CRT‐D patients (7.8%) of the JCDTR group. The rate was 1.9% at 1 year and 4.2% at 2 years in the New JCDTR group, and 4.5% at 1 year and 9.1% at 2 years in the JCDTR group (*p* = .002) (Figure 3C).

An adjusted hazard ratio (aHR) for appropriate ICD therapy, inappropriate ICD therapy and death from any cause was 0.76 (95% confidence interval [CI]: 0.59–0.98; *p* = .032), 0.24 (95% CI: 0.12–0.50; *p* < .0001) and 0.72 (95% CI: 0.55–0.94; *p* = .017) in CRT‐D patients of the New JCDTR group versus the JCDTR group (Table 3). Reduction in noncardiac death mainly contributed to the decrease in mortality of the latest CRT‐D patients as compared to earlier patients.

**TABLE 3:** Hazard ratios for events in CRT‐D recipients of the New JCDTR group versus the JCDTR group.

Events	Hazard ratio	95% CI	*p* value
Appropriate ICD therapy	0.76	0.59–0.98	.032
Inappropriate ICD therapy	0.24	0.12–0.50	<.0001
Death from any cause	0.72	0.55–0.94	.017
Heart failure death	0.85	0.58–1.26	.43
Sudden cardiac death	1.11	0.55–2.23	.77
Noncardiac death	0.38	0.23–0.63	.002

*Note*: Models were adjusted for the following covariates: age at enrollment, gender, indication, etiology, LVEF, NYHA class, QRS width, and hemoglobin and creatinine levels.

Abbreviations: CI, confidence interval; ICD, implantable cardioverter–defibrillator; LVEF, left ventricular ejection fraction.

## 4 DISCUSSION

The present study demonstrated that patients implanted with a CRT‐D over the latest years (the New JCDTR group) had a better survival than those in the earlier years (the JCDTR group) despite more comorbidities and aged population. We had expected that prognosis of the latest CRT‐D patients was better than that of the earlier with a reduction in heart failure death, because the revision of guidelines^11^, ^12^, ^13^ advocated selecting patients with LBBB morphology for CRT indication which was likely to reduce heart failure progression.^8^ However, the better prognosis was not attributable to decrease in heart failure death, but was yielded by a substantial decline in noncardiac death. Although ischemic CRT patients had a worse prognosis,^17^, ^19^, ^20^, ^21^ there was no difference in etiologies, that is ischemic versus nonischemic, between the JCDTR and New JCDTR groups. In terms of the decline in noncardiac death, spillover effects of COVID‐19 pandemic^22^ may have prevented other infections for example, pneumonia or influenza.^23^, ^24^, ^25^

Cardiovascular death significantly decreased in heart failure patients with advancing calendar year from 2000 to 2010 in Japan^26^ and from 1997 to 2016 in Danish,^27^ which was associated with increased use of guideline‐directed medical therapy. In the present study, treatment with ACEI/ARB remained at the same level with an average annual rate of 66% (Figure S1), which was lower than 74% of the Danish study and that of a multinational registry study.^28^ Further implementation of evidence‐based medications, with maximal tolerated or target dose,^29^ including the angiotensin receptor neprilysin inhibitor and sodium‐glucose cotransporter 2 (SGLT2) inhibitors would be required to reduce heart failure death in CRT‐D patients.

Both appropriate and inappropriate ICD therapies were reduced in the New JCDTR group with the rate of 15% and 1.5%, as compared to the JCDTR group with the rate of 20% and 5.7%, during an average follow‐up of 1.8 years (21 months). The difference in appropriate ICD therapy did not emerge until 1 year after device implantation, whereas that in inappropriate ICD therapy began early after device implantation (Figure 2A,B). Regarding appropriate ICD therapy, the rate in the New JCDTR group (15%) was lower than 22% in Conventional Therapy of the MADIT‐RIT trial during an average follow‐up of 1.4 years.^18^ Additionally, the rate of inappropriate ICD therapy in the New JCDTR group (1.5%) was equivalent to 2% in High‐Rate Therapy and 3% in Delayed Therapy of the MADIT‐RIT trial. It is likely that we have adopted the current recommended settings to reduce inappropriate ICD therapy over the latest years.^18^, ^30^ Besides, current recommendations for CRT implants with an emphasis on prolonged QRS width and LBBB morphology^11^, ^12^, ^13^ would have resulted in better left ventricular structural remodeling, which continued to occur during the first 6 months of CRT,^31^ with a time‐dependent reduction in the risk of life‐threatening ventricular arrhythmias.^32^, ^33^

Increased prevalence of cardiac sarcoidosis was a notable difference in underlying heart diseases of CRT‐D patients during the latest years, which is in agreement with an observation that the detection rate of cardiac sarcoidosis has increased markedly over the recent years.^34^ This is most likely as a result of the improved diagnostic methods and heightened diagnostic activity.^35^ In addition, we may be recognizing a risk of sudden cardiac death in patients presenting with cardiac sarcoidosis.^35^

There are several limitations to be considered in this study. First, this registry did not collect data regarding QRS morphology and location of the left ventricular lead. Second, device programming for ventricular arrhythmias was left on the discretion of attending physicians. However, participants in this cohort study have a certificate of Heart Failure Treatment with ICD and Cardiac Pacing endowed by the JHRS and Japanese Heart Failure Society (JHFS) and would be familiar with contemporary programming. Third, information regarding the presence or absence of nonsustained VT and atrial fibrillation, which is likely to affect outcomes, is not mandatory for the registration in the JCDTR. Therefore, the prevalence of atrial fibrillation in the JCDTR group is underestimated. Fourth, the rate of the patients who were followed was 23% in the JCDTR and 25% in the New JCDTR. In order to show the prognosis more accurately, we have to increase the follow‐up rate in this registry.

In conclusion, all‐cause mortality was reduced in CRT‐D patients implanted during the year 2018–2021 as compared to those during the year 2011–2015, with a significant decrease in noncardiac death. Inappropriate ICD therapy was also reduced early after device implantation, whereas reduction in appropriate ICD therapy emerged about 1 year after the implantation.

## CONFLICT OF INTEREST STATEMENT

Authors declare no conflict of interests for this article.

## ETHICS APPROVAL

The JCDTR and New JCDTR were approved in the Ethics Committee of Sapporo City General Hospital on May 16, 2018 (Approval No. H30‐057‐455).

## PATIENT CONSENT

Patient consent has been obtained in opt‐out manner in Sapporo City General Hospital.

## Supporting information


Figure S1.
