# Supplementary material for: Improved outcomes of cardiac resynchronization therapy with a defibrillator in systolic heart failure: Analysis of the Japan cardiac device treatment registry database
Source: J Arrhythm. 2023 Nov 14;40(1):30–7. doi: 10.1002/joa3.12952 (PMC10848589; doi:10.1002/joa3.12952)

**Supplemental Figure**

**Figure S1. Annual trends in number of CRT-D implant with prescription rates of guideline-recommended drugs registered in the JCDTR and New JCDTR.**

The absolute number of de novo CRT-D implant is given by orange bar. Blue, dark orange and gray lines indicate annual prescription rates of β-blocker, angiotensin converting enzyme inhibitor (ACEI) or angiotensin II receptor blocker (ARB) and mineral corticoid receptor antagonist (MRA), respectively.

**Figure S1**


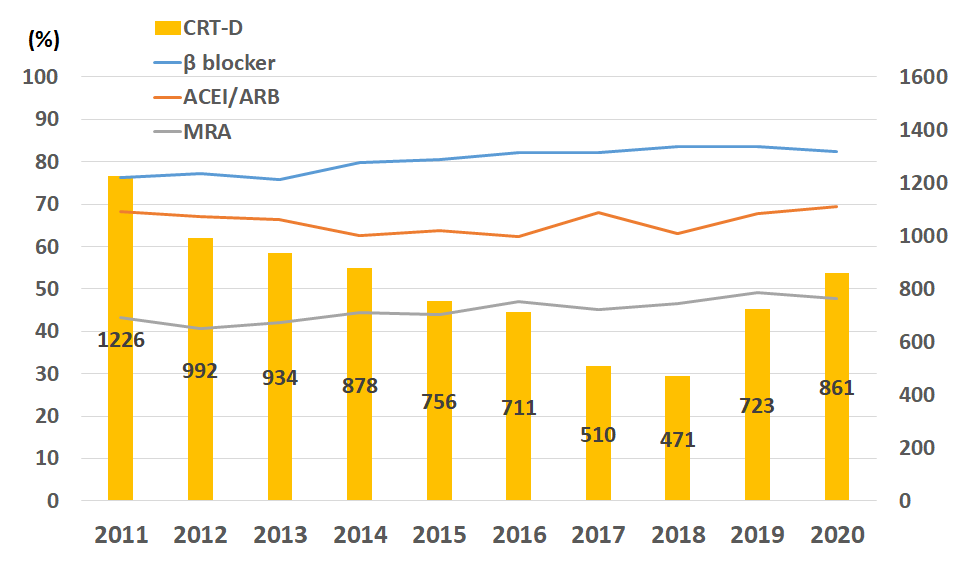

Supplement: Supplementary file 1 — Figure S1. [file JOA3-40-30-s001.docx]
